# The Utility of Artificial Intelligence Platforms for Post‐Operative Mohs Micrographic Surgery Questions: A Blinded Expert Panel Evaluation

**DOI:** 10.1111/ijd.70232

**Published:** 2026-01-07

**Authors:** Eva M. Shelton, Janmesh Patel, Murad Alam, Anna Bar, M. Laurin Council, Thomas A. Rohrer, Emily Ruiz, Ashley Wysong, Siegrid S. Yu, Stanislav N. Tolkachjov

**Affiliations:** ^1^ Department of Dermatology University of Wisconsin‐Madison Madison Wisconsin USA; ^2^ School of Medicine and Public Health University of Wisconsin‐Madison Madison Wisconsin USA; ^3^ Department of Dermatology Northwestern University Feinberg School of Medicine Chicago Illinois USA; ^4^ Department of Dermatology Oregon Health and Science University Oregon Portland USA; ^5^ Division of Dermatology, Department of Medicine Washington University School of Medicine St. Louis Missouri USA; ^6^ SkinCare Physicians Chestnut Hill Massachusetts USA; ^7^ Department of Dermatology Brigham and Women's Hospital Boston Massachusetts USA; ^8^ Department of Dermatology Mayo Clinic Jacksonville Florida USA; ^9^ Department of Dermatology University of California San Francisco San Francisco California USA; ^10^ Epiphany Dermatology Dallas Texas USA; ^11^ Department of Dermatology University of Texas at Southwestern Dallas Texas USA; ^12^ Texas A&M School of Medicine Dallas Texas USA; ^13^ Division of Dermatology Baylor University Medical Center Dallas Texas USA

**Keywords:** artificial intelligence, infection, Mohs surgery, post operative care, skin cancer, wound care

Artificial intelligence (AI) and large language models (LLMs) are increasingly used by Mohs surgery patients, who often seek additional online guidance for wound care, pain control, and infection recognition despite standard discharge instructions [1, 2]. Prior studies examined AI in dermatologic education [3, 4], but the quality of LLM‐generated responses to postoperative Mohs questions remains uncertain. This study evaluates their accuracy, appropriateness, and sufficiency.

Three board‐certified Mohs surgeons (MA, AW, SY) finalized twelve commonly asked postoperative questions. These were posed to ChatGPT‐4o, Gemini 2.0 Flash, and LLaMA 4 in separate memory‐cleared sessions. Responses were compiled into a blinded, randomized Qualtrics survey reviewed by eight Mohs surgeons (including the original three). Reviewers rated each response on a 5‐point Likert scale for sufficiency, accuracy, and appropriateness, and flagged deficiencies including omissions, ambiguities, or inaccuracies. Mean scores (±SD) were calculated, and one‐way ANOVA compared ratings (*p* < 0.05) (Table 1). Inter‐rater reliability was assessed using Fleiss kappa.

**TABLE 1:** Mean ratings (standard deviation) of AI‐generated responses to postoperative Mohs surgery questions across three domains: Sufficiency, accuracy, and appropriateness. Twelve questions were posed to ChatGPT‐4o, Gemini 2.0, and LLaMA 4. Eight board‐certified Mohs surgeons rated each response on a 5‐point Likert scale (1 = poor, 5 = excellent). Mean (SD) scores are shown per question, model, and evaluation domain. One‐way ANOVA was used to compare model performance for each domain.

Survey questions	Sufficiently addresses patient concern			Accuracy of response			Appropriateness for patient‐facing setting		
	GPT‐4o	Llama 4	Gemini 2.0	GPT‐4o	Llama 4	Gemini 2.0	GPT‐4o	Llama 4	Gemini 2.0
What can I do for pain or discomfort after Mohs surgery, and can I take ibuprofen, Tylenol, or aspirin for relief?	4.13 (0.35)	3.63 (0.92)	4.13 (0.83)	4.13 (0.64)	3.75 (0.71)	4.13 (0.83)	4 (0.93)	3.88 (0.99)	4.13 (0.83)
What should I do if my wound starts to bleed?	3.88 (0.35)	3.75 (1.04)	4.38 (0.74)	4.29 (0.76)	3.75 (1.04)	4 (0.93)	4 (0.76)	4 (0.76)	4.25 (0.71)
How do I know if I need to call my doctor for a post‐operative concern?	3.88 (0.64)	3.88 (0.83)	4.63 (0.74)	4.25 (0.71)	3.88 (0.83)	4.5 (0.76)	3.88 (0.99)	4 (0.53)	4.38 (0.74)
How should I take care of my wound after surgery?	3.38 (1.06)	4 (0.53)	4.38 (0.52)	3.38 (0.74)	4 (0)	4.13 (0.64)	3.5 (0.93)	4 (0.53)	4.25 (0.71)
Will my stitches need to be removed? When?	4 (0.53)	3.38 (0.92)	4.25 (0.89)	4.13 (0.64)	4 (0.76)	3.88 (0.83)	4.13 (0.64)	4 (0.76)	4.13 (0.64)
How often should I change my bandage?	4.25 (0.71)	2.75 (1.16)	4.13 (0.83)	4 (0.76)	3.63 (0.74)	4 (0.93)	4.25 (0.71)	3.63 (0.92)	4.25 (0.89)
What can I do to minimize scar formation?	4.13 (0.64)	3.88 (0.64)	4.25 (1.04)	3.88 (0.64)	3.5 (0.53)	4 (0.93)	4 (0.76)	3.57 (0.79)	4.13 (1.13)
When can I get the wound wet, shower, or take a bath?	4 (0.53)	3.38 (0.52)	4 (0.76)	4.25 (0.89)	4 (0.53)	4.13 (0.83)	4.25 (0.71)	4.13 (0.64)	4.38 (0.74)
What are the signs of infection I should watch for, like redness, warmth, swelling, odor, or unusual drainage?	4.25 (0.71)	4.5 (0.53)	4.63 (0.74)	4.38 (0.74)	4.5 (0.53)	4.63 (0.74)	4.38 (0.74)	4.5 (0.53)	4.63 (0.74)
How long will numbness around the wound last?	4.5 (0.53)	3.25 (0.71)	4.63 (0.52)	4.63 (0.52)	3.75 (0.71)	4.63 (0.52)	4.5 (0.76)	3.75 (1.04)	4.63 (0.52)
When can I resume normal activities like exercise or work?	3.88 (0.64)	3.75 (0.71)	4.38 (0.74)	4.13 (0.64)	4 (0.76)	4.38 (0.74)	4.25 (0.89)	4.13 (1.13)	4.25 (0.89)
How much scarring should I expect, and will it fade over time?	4 (0.76)	3.75 (1.04)	3.88 (0.83)	3.88 (0.83)	4.25 (0.71)	4.25 (0.71)	4.13 (0.83)	4.13 (0.83)	4.13 (0.83)
One‐Way ANOVA	*p* < 0.001			*p* = 0.018			*p* = 0.023		

Sufficiency: Gemini 2.0 achieved the highest sufficiency ratings (mean 4.30), followed by GPT‐4o (4.02) and LLaMA 4 (3.66). Sufficiency was strongest for guideline‐driven questions with clear answers, such as when to call the doctor (Gemini 4.63) and signs of infection (Gemini 4.63), which all models scored above 4. In contrast, more nuanced questions requiring individualized guidance, such as general wound care and scar management, scored lower, with LLaMA 4 performing worst (*p* < 0.001) (Table 1, Figure 1).

**FIGURE 1:**
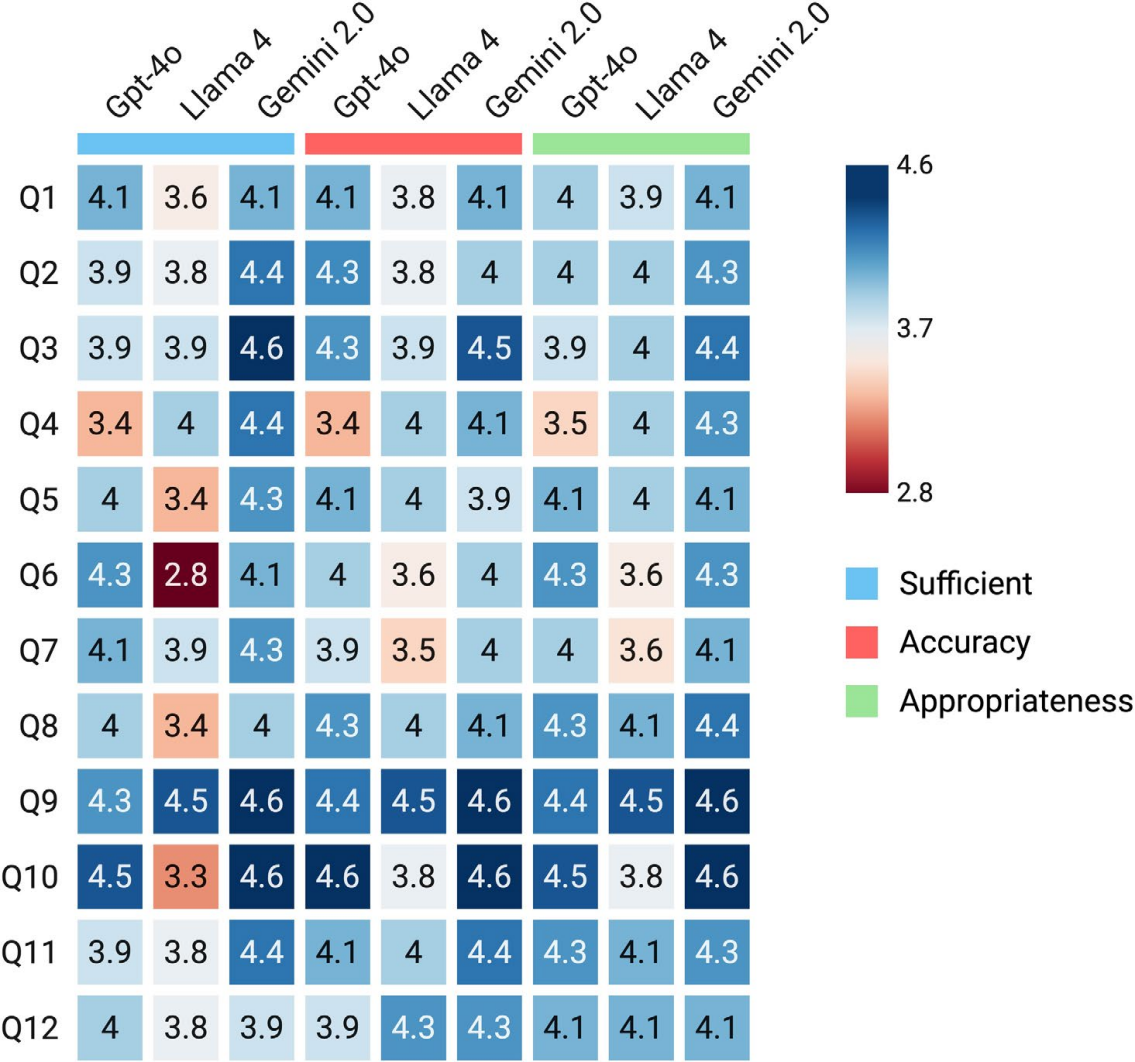
Heat map visualization of AI model performance across sufficiency, accuracy, and appropriateness domains. Each colored cell represents the mean score for a given question, model, and domain. Higher scores (darker blue) indicate stronger performance. Questions are labeled Q1–Q12, corresponding to the same items in the accompanying table. Domains are color‐coded: Blue (sufficiency), red (accuracy), green (appropriateness).

Accuracy: All models demonstrated accuracy above 3.5. Gemini 2.0 (mean 4.22) and GPT‐4o (4.11) performed comparably, with GPT‐4o achieving the highest scores for how long numbness lasts (4.63) and when to get the wound wet (4.25). Accuracy was strongest for infection‐related questions and lower for scar management and stitch removal. Differences were significant (*p* = 0.018), with Gemini 2.0 and GPT‐4o outperforming LLaMA 4 (3.92) (Table 1, Figure 1).

Appropriateness: Appropriateness ratings were consistently high. Gemini 2.0 scored highest (4.30), followed by GPT‐4o (4.11) and LLaMA 4 (3.98). Gemini performed best for infection‐related topics. Scores overall declined when responses were overly technical or too vague. Differences were statistically significant (*p* = 0.023) (Table 1, Figure 1).

Deficiency: Eight surgeons evaluated 36 responses (12 × 3) and could flag multiple deficiencies per response; counts reflect total flags across responses and reviewers (139 total deficiencies). Omissions were the most frequent (47% (65/139)), followed by ambiguous guidance and medically inaccurate information (each 22% (31/139)). Unsafe advice (7% (9/139)) and poor readability were uncommon (2% (3/139)). LLaMA 4 accounted for most omissions (62% (40/65)), and over half of ambiguous guidance (61% (19/31)). Inaccuracies were similar across models. (Supporting Information Table S1 is available via Mendeley: https://data.mendeley.com/datasets/kxwzmgx62v/2).

Across all three LLMs, postoperative Mohs responses were generally accurate and appropriate, although sufficiency varied. Gemini 2.0 and GPT‐4o performed better than LLaMA 4, and all models scored highest on structured, guideline‐driven topics such as signs of infection. Infection‐related questions likely performed well because the expected clinical features, red flag symptoms, and recommended actions are consistent across surgeons and align with well‐established principles of postoperative care. More nuanced areas such as wound care routines and scar management received lower ratings, which is consistent with their dependence on surgeon preference, reconstruction type, and individualized patient factors. LLMs cannot account for this variability, which may explain their lower performance on questions that require tailoring or detailed interpretation [5].

Although we did not formally report inter‐rater reliability, reviewers demonstrated low agreement in their ratings. This reflects natural variability in how Mohs surgeons counsel patients about wound care and postoperative expectations rather than a limitation of the methodology. Because postoperative guidance is individualized and shaped by wound type, reconstruction choice, and surgeon practice patterns, it is expected that expert evaluators apply different thresholds for sufficiency, accuracy, and appropriateness. This variability also helps explain why LLM responses were often judged incomplete and reinforces that AI generated answers cannot replace individualized postoperative communication [3, 4].

The purpose of evaluating LLM generated responses is not to promote AI as a substitute for surgeon guidance. Many Mohs patients, especially those from remote areas, already consult AI tools after surgery despite receiving written instructions and having direct phone access. Characterizing the quality of the information they encounter is therefore clinically relevant. Our findings show that while the answers are generally safe and reasonable, omissions and ambiguous guidance are common, especially for topics that require individualized clinical judgment. These insights may help surgeons anticipate areas of confusion and refine patient education materials. Our findings do not suggest that LLMs are currently sufficient for clinical use, and we did not evaluate regulatory considerations or patient outcomes. Any potential usefulness is limited to supplemental education and must remain under direct surgeon oversight.

## Funding

The authors have nothing to report.

## Conflicts of Interest

Dr. Tolkachjov is an investigator and a speaker for CASTLE Biosciences, Kerecis, Regeneron, and Boehringer Ingelheim. The authors do not have any relevant conflicts of interest to declare.

## Data Availability

The data that support the findings of this study are available from the corresponding author upon reasonable request.
